# Retraction Note: Characteristic differences in the minimental state examination used in Asian countries

**DOI:** 10.1186/s12883-024-03698-w

**Published:** 2024-06-07

**Authors:** Yong S. Shim, Dong Won Yang, Hee-Jin Kim, Young Ho Park, SangYun Kim

**Affiliations:** 1 Department of Neurology, College of Medicine, Bucheon St. Mary’s Hospital, The Catholic University of Korea, Seoul, Republic of Korea; 2 Department of Neurology, Seoul St. Mary’s Hospital, College of Medicine, The Catholic University of Korea, Seoul, Republic of Korea; 3 Department of Neurology, College of Medicine, Hanyang University, Seoul, Republic of Korea; 4 Department of Neurology, Clinical Neuroscience Center, Seoul National University College of Medicine, Seoul National University Bundang Hospital, Seoul, Republic of Korea; 5 82, Gumi-ro 173 beon-gil, Bundang-gu, Seongnam-si, Gyeonggi-do, 13620 South Korea; 6 327 Sosa-ro, Wonmi-gu, Bucheon-si, Gyeonggi-do, 14647 South Korea

**Retraction Note: BMC Neurol 17, 141 (2017)**

10.1186/s12883-017-0925-z

The Editor has retracted this article because the authors did not obtain permission to reproduce the MMSE scale. The content of this article has been removed for legal reasons. Yong S. Shim, Dong Won Yang and SangYun Kim disagree with this retraction. Hee-Jin Kim and Young Ho Park have not responded to correspondence from the Editor about this retraction.

